# Emetine and Its Derivatives as Anticancer Agents Targeting the PI3K/AKT/mTOR Pathway: A Narrative Review of Mechanisms, Pharmacology, and Clinical Insights

**DOI:** 10.1111/jcmm.71328

**Published:** 2026-08-23

**Authors:** Mst. Ismatara Khatun, Samy Selim, Asif Hassan Malik, Md. Sakib Al Hasan, Abul Bashar Ripon Khalipha, Noshin Tasnim Yana, Mohammad Y. Alshahrani, Md. Arif Hossain, Imam Hossen Rakib, Anike Chakrabarty, Emon Mia

**Affiliations:** ^1^ Department of Pharmacy, Faculty of Biological Science and Technology Jashore University of Science and Technology Jashore Bangladesh; ^2^ Department of Clinical Laboratory Sciences, College of Applied Medical Sciences Jouf University Sakaka Saudi Arabia; ^3^ Department of Chemistry York College of The City University of New York (CUNY) Jamaica New York USA; ^4^ Department of Pharmacy Gopalganj Science and Technology University Gopalganj Bangladesh; ^5^ Alor Dishari Research Organization Dhaka Bangladesh; ^6^ Central Labs King Khalid University Abha Saudi Arabia; ^7^ Department of Clinical Laboratory Sciences, College of Applied Medical Sciences King Khalid University Abha Saudi Arabia; ^8^ Department of Biotechnology, Faculty of Engineering and Science University of Greenwich Kent UK

**Keywords:** Anticancer, Emetine, Literature review, PI3K‐AKT‐mTOR pathway, toxicity

## Abstract

Emetine (EMT), a naturally occurring tetrahydroisoquinoline alkaloid, shows diverse biological actions, including anticancer effects. Despite existing studies, the role of EMT derivatives in cancer, particularly in relation to the PI3K/AKT/mTOR signalling pathway, remains largely underexplored. This review explores the potential of EMT in targeting PI3K/AKT/mTOR molecular pathways across different cancer types. Data were collected from reliable and well‐established sources, including PubMed, Scopus, Wiley Online, Web of Science, ScienceDirect, and Google Scholar. Findings showed that EMT derivatives suppress PI3K‐AKT‐mTOR pathway activity in multiple cancers such as liver, gastric, acute myeloid leukaemia (AML), and brain, with IC_50_ values ranging from 0.0244 to 1 μM. Beyond its anticancer activity, EMT showed broad pharmacological relevance, including antiviral, antiparasitic, and contraceptive activities. Preclinical assessments suggest preferential cytotoxicity toward cancer cells at low concentrations; however, known dose‐dependent adverse effects, including cardiotoxicity and emetic responses at higher doses, necessitate careful dose optimization before clinical translation. Clinical evidence remains insufficient, and EMT should be considered a preclinically active compound warranting further rigorous translational investigation.

## 1 Introduction

Cancer is the uncontrolled growth and development of cells in the body, and is one of the foremost reasons for deaths throughout the world [1]. According to the International Agency for Research on Cancer (IARC), there were close to 20 million new cases of cancer in the year 2022 alongside 9.7 million deaths from cancer, with demographics‐based predictions indicating that the number of new cases of cancer will reach 35 million by 2050 [2]. Environmental, exogenous and endogenous factors, as well as individual factors including genetic predisposition, contribute to the development of cancer. In addition, smoking, excessive alcohol consumption, diet, and reproductive behaviour are important for the development of malignant neoplasia in the human population [3].

However, several pathways are involved in cancer progression, and one of the key pathways implicated in cancer development is the PI3K/AKT/mTOR [4]. The PI3K/AKT/mTOR signalling regulates cell proliferation, differentiation, cellular metabolism, and cytoskeletal reorganization leading to apoptosis and cancer cell survival [5]. Phosphoinositide 3‐kinase (PI3K) is a major signalling component downstream of growth factor receptor tyrosine kinases (RTKs), and PI3K catalyses the production of the lipid second messenger phosphatidylinositol‐3,4,5‐triphosphate (PIP3) at the cell membrane. PIP3, in turn, contributes to the recruitment and activation of a wide range of downstream targets, including the serine–threonine protein kinase AKT [6].

The PI3K/AKT/mTOR signalling pathway is hyperactivated or altered in many cancer types and regulates a broad range of cellular processes including survival, proliferation, growth, metabolism, angiogenesis and metastasis [7] and copponents of this pathway are frequently abnormal in a variety of tumours, making them an attractive target for anti‐cancer therapy [8]. Numerous PI3K/AKT/mTOR pathway inhibitors are extensively studied; some are used clinically, but most of these drugs are undergoing clinical trials [9]. The most common side effect associated with PI3K/AKT/mTOR pathway inhibitors including, hyperglycaemia [10], pneumonitis [9], stomatitis [11], skin rash [12], mood disturbance [13], gastrointestinal effects [14]. As a result, further investigation is needed for safe, effective therapeutic application targeting PI3K/AKT/mTOR inhibitor pathway. Natural products may provide pharmacologically relevant bioactivities. However, their efficacy and safety require rigorous experimental and clinical validation. As far as cancer is concerned, naturally occurring compounds have been reported to prevent tumorigenesis [15]. Several natural products and its derivatives like Quercetin [16], Arctigenin [17], isoflavones [18], curcumin [19], Fisetin [20] that induce autophagy by inhibiting PI3K/Akt/mTOR signalling [21].

Natural compounds (NCD) have gained significant attention in anticancer research due to their multitargeted actions and ability to modulate key oncogenic pathways involved in tumour progression [22]. Polyphenols have demonstrated promising therapeutic potential in pancreatic cancer by regulating multiple molecular targets and signalling pathways associated with tumour growth and survival [23]. Flavone derivatives and related flavonoids have shown strong antagonistic activity against oestrogen receptor‐α (ER‐α), indicating their relevance in hormone‐dependent cancer therapy [24]. Plumbagin, a naturally derived naphthoquinone, has exhibited potent anticancer activity through regulation of apoptosis and inhibition of cancer cell proliferation [25]. Curcumin combined with plumbagin has shown strong inhibitory effects on the PI3K/Akt/mTOR signalling pathway, supporting their potential as combination anticancer agents [26]. Phenolic acids have been reported to regulate multiple molecular targets in ovarian cancer, further supporting their therapeutic relevance in gynaecological malignancies [27]. Synergistic combinations such as luteolin and ferulic acid have demonstrated enhanced anticancer effects through simultaneous targeting of multiple oncogenic pathways in glioblastoma models [28]. Novel drug delivery systems and synthetic derivatives, including propofol‐linoleic acid formulations and thiosemicarbazone compounds, have demonstrated enhanced anticancer efficacy in preclinical models [29, 30].

The use of botanical plants, herbs, fungi, seeds as medicines predates recorded history and represents the most significant direct antecedent to modern medicine [31]. Medical indications of NCD and related drugs, including anticancer [32]. The main goals of including NCD in cancer chemotherapies are to widen the therapeutic window of the chemotherapeutic drugs and to decrease the occurrence of chemotherapy resistance [33]. However, EMT, isolated from *Psychotria ipecacuanha* , is a tetrahydroisoquinoline alkaloid [34, 35] and it is considered as a potential anti‐cancer agent for various human cancers [36], including gastric cancer (GC) [37], lung cancer [36], oesophageal cancer [38] and endometrial cancer (Bredholt et al., 2015). In addition, it has been demonstrated that EMT showed a wide range of activity including anti‐tumour [39], anti‐SARS‐CoV‐2 [40], and treating pulmonary arterial hypertension [41], acute intestinal and extraintestinal amoebiasis [40].

In this literature evidence highlights that EMT and its derivatives exhibited anticancer effect by inhibiting PI3K/AKT/mTOR signalling pathway. The main purpose of this study is to compile and critically evaluate the preclinical and limited clinical evidence on EMT and its derivatives as potential anticancer agents targeting the PI3K/AKT/mTOR pathway, and to identify mechanistic and translational gaps that warrant further investigation.

## 2 Methodology

### 2.1 Literature Search Strategy

This narrative review synthesizes published evidence identified through a non‐exhaustive literature search conducted across multiple scientific databases including, PubMed, ScienceDirect, Google Scholar, Web of Science, Scopus, Wiley Online. The term ‘emetine derivatives’ was combined with the following keywords that were used to utilise this study including: ‘cancer’, ‘tumour’, ‘anticancer activity’, ‘antitumor activity’, ‘human cancer’, ‘PI3K‐AKT–mTOR pathway’, ‘natural sources’, ‘cytotoxic activity’, ‘biological activities’, ‘pharmacological activities’, ‘pharmacological effects’, ‘pharmacokinetics’, ‘chemical features’, ‘in vivo studies’ and in vitro studies. In this search parameters no time and language restrictions were imposed. These studies were thoroughly evaluated with the sources, dosage, concentration, test system, suggested action mechanism, overall conclusion was included.

### 2.2 Inclusion Criteria

The studies included for this literature were selected based on the following criteria:

1. Research conducted in vivo, ex vivo, or in vitro from laboratory animals using their tissues or cells.
2. Studies on the anticancer effects of EMT and its bioactive compounds via the PI3K‐AKT‐mTOR pathway.
3. Additionally, studies were included either does not or does propose mechanisms of action.

### 2.3 Exclusion Criteria

Studies were excluded in this review if they met any following criteria:

1. Repetitive data, abstract, title that did not match the guideline for inclusion criteria.
2. Other studies have not found that EMT solves the current issue.
3. Articles published in languages other than English.
4. Research that is unpublished or not available in full text.
5. Case studies, analyses, reviews, and letters.

## 3 Results and Discussion

### 3.1 Literature Review of Anticancer

This narrative review synthesizes reported evidence on the inhibitory effects of EMT and its derivatives in various cancer cell lines, focusing on their modulation of the PI3K/AKT/mTOR signalling pathways, which play a key role in regulating cell survival, proliferation and growth. This review also evaluates multiple cancer types, experimental models, and tested concentrations.

#### 3.1.1 Acute Myeloid Leukaemia

AML is a heterogeneous group of leukaemias that result from clonal transformation of haematopoietic precursors through the acquisition of chromosomal rearrangements and multiple gene mutations [42]. The PI3K/AKT/mTOR pathway is central for haematopoietic cells and regulates crucial functions such as proliferation, differentiation, and survival. This pathway seems to be constitutively activated in 60% of AML patients and this activation seems to be associated with decreased overall survival [43]. However, as mTORC1 activation is independent of PI3K/AKT in AML, dual PI3K and mTOR inhibitors may induce apoptosis in blast cells [44]. These studies exhibit that EMT inhibit PI3K/AKT/mTOR signalling pathways in AML cancer cells with concentrations of 0.5–2 μM [45]. Although EMT demonstrated suppression of PI3K/AKT/mTOR signalling in AML models, the majority of available studies primarily evaluated phosphorylation status rather than direct molecular target engagement. Therefore, it remains unclear whether EMT directly inhibits components of the pathway or exerts indirect upstream effects through cellular stress responses. Moreover, the heterogeneity of AML subtypes and the limited number of animal studies reduce the generalizability of these findings. These findings indicate that EMT modulates PI3K/AKT/mTOR signalling in AML cell lines and xenograft models at low micromolar concentrations. The xenograft model used immunodeficient NSG mice, which do not fully recapitulate the human immune microenvironment, and formal IC_50_ value for therapeutic potential.

#### 3.1.2 Gastric Cancer

GC is a multifunctional disease that is the fourth leading cause of death worldwide [46]. Alterations of the PI3K/AKT/mTOR pathway are widely observed in numerous tumours, including GC [47]. The catalytic subunit PIK3CA gene is mutated at a high frequency, and the PIK3R3 gene, which encodes the PI3K regulatory subunit p55γ, was found to be significantly upregulated in gastric tumour specimens [48]. In addition, the loss of PTEN is associated with GC initiation and development [46]. In GC studies, it was revealed that EMT demonstrated notable inhibitory activity against GC cell growth in experimental models, with IC_50_ values of 0.0497 and 0.0244 μM respectively and also suppressed PI3K/AKT signalling in MGC803 and HGC‐27 cells [37]. Importantly, the observed nanomolar potency in GC models suggests strong biological activity; however, these findings should be interpreted cautiously because the studies were conducted predominantly in established cell lines lacking tumour microenvironment complexity. Additionally, resistance mechanisms associated with PI3K pathway mutations remain insufficiently explored. These findings suggest that EMT could be a mechanistically relevant finding in GC; however, both cell lines tested (MGC803, HGC‐27) represent intestinal‐type GC, and it remains to be determined whether these effects extend to diffuse‐type or drug‐resistant GC subtypes. In vivo dose–response characterization is needed before therapeutic feasibility can be assessed.

#### 3.1.3 Glioblastoma/Glioblastoma Multiforme

Glioblastoma multiform (GBM) is the most common malignant glioma of all the brain tumours [49]. The PI3K/AKT/mTOR signalling pathway promotes tumour growth and progression by regulating cell cycle activities and, upon phosphorylation by AKT, activated mTOR causes cyclin D1 to bind to cyclin dependent kinase (CDK) and initiate cell division [50]. In addition, PTEN depletes levels of PIP3, and its mutations are associated with 20%–40% of malignant gliomas [51]. This study reported that EMT reduced PI3K signalling at concentrations of 100 nM [52]. Another study revealed that EMT inhibited mTOR signalling in T98G cells at concentrations of 10 μM [53]. These findings suggest that EMT modulates PI3K and mTOR signalling in GBM cell line models; however, the concentration ranges used (up to 10 μM) may exceed pharmacologically achievable levels in vivo, and independent replication is needed before therapeutic conclusions can be drawn.

#### 3.1.4 Hepatocellular Carcinoma

Hepatocellular carcinoma (HCC) is a heterogeneous tumour with diverse phenotypic and genetic characteristics, and its tumorigenesis involves a range of molecular mechanisms [54]. PI3K is especially highly expressed in HCC tumour tissue, and the upregulation of PIK3CA was associated with HCC proliferation and negatively correlated with apoptosis [55]. AKT1 mediated phosphorylation of mTORC2 is crucial for triggering hepatocarcinogenesis and AKT2 also promotes cell proliferation and invasion [55, 56]. In addition, PTEN, the tumour suppressor that inhibits the mTOR pathway, is inactivated in approximately half of HCC tumours [57]. In an in vitro investigation on Huh7 and Hep3B cells, EMT downregulates AKT signalling [58]. These results indicate that EMT suppresses AKT signalling in HCC cell lines in vitro. While preliminary, these findings provide a mechanistic rationale for further investigation. However, in vivo confirmation and dose–response characterization in HCC models are needed before any therapeutic conclusions can be drawn.

#### 3.1.5 Lung Cancer

Deregulation of PI3K/AKT/mTOR pathway is involved in lung tumorigenesis [59]. Upregulation of the AKT and mTOR pathway has also been illustrated in significant proportions of Non‐small cell lung cancer (NSCLC) tumours [60]. In lung cancer, PI3K activation frequently occurs in response to activating mutation RTK, amplification of PI3K, inactivation of PTEN, mutational activation of the PIK3CA gene [61]. PI3KCA and AKT1 mutations have been observed in 2%–5% and 1%–2% of NSCLC, respectively [62]. The absence of PTEN leads to unrestricted PI3K/AKT/mTOR pathway activation and a loss of PTEN expression indicates a poor prognosis [63]. An in vitro study demonstrated that EMT (0–3 μM) suppressed the PI3K/AKT pathways in A549 and H1299 cells with IC_50_ values of 0.243 and 1 μM, respectively [36]. In another investigation, it was shown that EMT downregulates PI3K signalling in the T790M cell line [64]. Furthermore, EMT at concentrations of 10 μM inhibited AKT signalling both in vivo and in vitro [65]. Collectively, the available evidence supports EMT‐mediated pathway suppression in lung cancer models, but the mechanistic consistency across studies remains variable. Some investigations report predominant effects on AKT phosphorylation, whereas others suggest upstream receptor‐level modulation, indicating that EMT may exert pleiotropic rather than pathway‐specific anticancer actions. These results suggest that EMT was reported to inhibit critical signalling pathways in experimental lung cancer models at low micromolar concentrations. These findings are mechanistically informative but require validation in additional preclinical systems, including patient‐derived xenograft models, before the anticancer potential of EMT in lung cancer can be more broadly assessed.

Collectively, current preclinical findings indicate that EMT and its derivatives possess promising anticancer activity through modulation of the PI3K/AKT/mTOR signalling pathway across several cancer models. Nevertheless, these findings are derived primarily from in vitro studies and limited animal investigations. The currently available evidence does not yet establish clinical efficacy or safety in humans. Important translational limitations include insufficient pharmacokinetic characterization, narrow therapeutic index, potential cardiotoxicity, limited tumour selectivity data, and lack of large‐scale clinical trials. Therefore, EMT should presently be regarded as an experimental preclinical candidate rather than a clinically validated anticancer therapy.

Current evidence suggests that EMT and its derivatives interfere with multiple upstream and downstream components of the PI3K/AKT/mTOR axis. Mechanistically, EMT appears to reduce receptor tyrosine kinase‐mediated signalling, thereby limiting PI3K activation and subsequent AKT phosphorylation. In several cancer models, EMT‐induced suppression of AKT signalling was associated with reduced mTORC1 activity, decreased protein synthesis, impaired cell‐cycle progression, and induction of apoptotic pathways.

**FIGURE 1:**
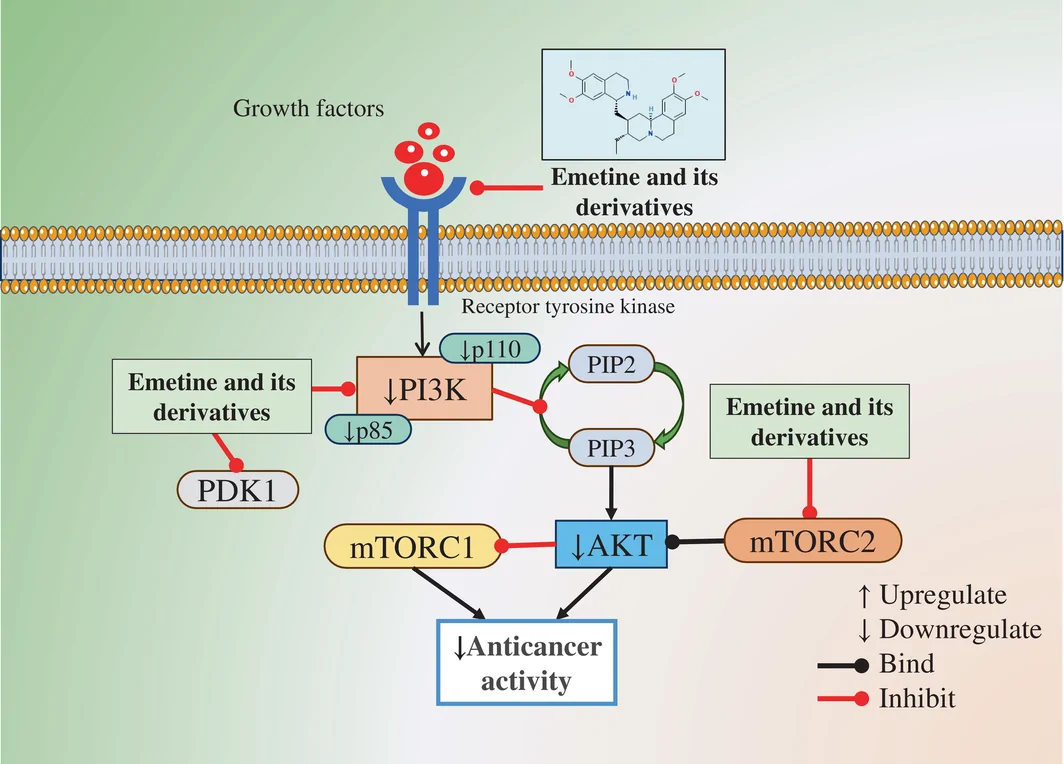
Anticancer mechanism of emetine and its derivatives. Emetine and its derivatives exert anticancer effects primarily by inhibiting the PI3K/AKT/mTOR signalling cascade initiated by receptor tyrosine kinases (RTKs). They suppress phosphoinositide 3‐kinase (PI3K; p110 catalytic and p85 regulatory subunits), preventing the conversion of phosphatidylinositol‐4,5‐bisphosphate (PIP2) to phosphatidylinositol‐3,4,5‐trisphosphate (PIP3), thereby reducing AKT (protein kinase B) activation. Inhibition of upstream regulators such as phosphoinositide‐dependent kinase‐1 (PDK1) and both mechanistic target of rapamycin complexes (mTORC1 and mTORC2) further attenuates survival and proliferative signalling. Collectively, this multi‐level blockade leads to downregulation of oncogenic signalling and suppression of cancer cell growth.

EMT may also influence pathway regulation indirectly through oxidative stress generation, HIF‐1α destabilization, mitochondrial dysfunction, and translational inhibition. These effects could converge on PI3K/AKT/mTOR signalling and contribute to reduced tumour cell survival. Importantly, most currently available studies rely heavily on western blot‐based phosphorylation analyses, while direct kinase‐binding assays, proteomic profiling, and genetic validation studies remain limited. Consequently, whether EMT acts as a direct pathway inhibitor or as a broader cellular stress modulator remains incompletely resolved. All of EMT's anticancer effects are presented in Table 1. And the anticancer mechanism of EMT is depicted in Figure 1.

**TABLE 1:** Mechanism of anticancer activity of Emetine and its derivatives through PI3K‐AKT–mTOR.

Compound name	Cancer type	Experimental model/cell lines	Tested concentrations (R/A)	IC_50_	Mechanisms/results	References
EMT	GC	MGC803 xenograft model, HGC‐27, in vivo	—	0.0497 and 0.0244 μM respectively	↓PI3K/AKT	[37]
EMT	Lung cancer	A549 and H1299 cancer cell lines, in vitro	0–3 μM	0.243 μM and 1 μM respectively	↓PI3K/AKT	[36]
Emetine dihydrochloride	Lung cancers	T790M cancer cell line, in vitro	—	—	↓PI3K	[64]
EMT	AML	Xenograft model in NSG mice, in vivo (*n* = 6)	0.5–2 μM	—	↓PI3K/AKT/mTOR	[45]
Emetine dihydrochloride	HCC	Huh7 and Hep3B cells, in vitro	—	—	↓AKT	[58]
EMT	GBM	U87, U138 and U373 cancer cell lines, in vitro	100 nM	—	↓PI3K/AKT	[52]
EMT	Lung cancer	NSCLC cancer cell line, in vitro	10 μM	—	↓AKT	[65]
EMT	Brain cancer	T98G cancer cell line, in vitro	10 μM	—	↓mTOR	[53]

*Note:* ↑: increase/activation/upregulation/stimulation; ↓: decrease/inhibition/downregulation.

Abbreviations: AKT, protein kinase B; AML, acute myeloid leukaemia; GBM, glioblastoma; GC, gastric cancer; HCC, hepatocellular carcinoma; mTOR, mechanistic target of rapamycin; PI3K, phosphoinositide 3‐kinase.

### 3.2 Pharmacological Relevance

Several studies demonstrated that, EMT used for treating various diseases condition because of its multiple therapeutic actions like antiviral, anticancer, antiparasitic and contraceptive activities [66]. Treatment with EMT inhibited pulmonary artery smooth muscle cells proliferation and also significantly reduced HIF‐1α and HIF‐2α and Rho‐kinase activity in PAH‐PASMCs. In addition, EMT with 0.05 mg/kg/day in male SD rats, reduced RV systolic pressure and RV hypertrophy [67]. In another investigation, it was observed to reduce IE1/2, UL44 and pp65 proteins and significantly inhibit human Cytomegalovirus (HCMV) replication by EMT (75 nM). Furthermore, EMT induces an interaction between MDM2 and RPS14 in infected cells [68]. Against COVID‐19 EMT at 1 mg/kg concentrations potently inhibit SARS‐CoV‐2 virus replication and reduce IL‐6, TNF‐α protein level [69]. EMT (32 and 64 μg/μL) exhibited a neurophysiological effect by suppressing amino acid synthesis. Besides that, EMT inhibited theta power profile [70]. In another investigation confirmed that EMT dihydrochloride at 0.01–10 μM concentrations showed a potent antiviral activity by inhibiting synthesis viral RNA and DENV replication [71]. The findings also exhibited that, EMT mitigate zika virus infection. At 2 mg/kg/day concentrations EMT decreased NS1 protein level and also inhibited ZIKV replication and NS5 polymerase activity. Along with this, against Ebola virus, EMT (1 mg/kg/day) suppressed lysosomal function and decreased viral entry and leading to inhibition of autophagy [72]. In a further study, EMT (0.03–1.0 mM) showed an emetogenic action by increasing Fos‐ir cells and changed in the excitability of brainstem neurons that induce emesis or nausea with an IC_50_ value 0.348 mM [73]. Administration of EMT dihydrochloride (0.002–2 mg/kg) to NOD mice reduced TNF levels in Type 1 diabetes mellitus [74]. In Enterovirus‐A71, EMT (0.20 mg/kg) reduce viral loads and inhibiting viral IRES‐driven translation [75]. EMT has a wide range of pharmacological activities, making it a versatile candidate for further research toward developing novel treatment strategies. Pharmacological activities of EMT are given in Table 2.

**TABLE 2:** Pharmacological relevance of emetine and its derivatives.

Compound name	Diseases/effect name	Dose/concentration	Mechanism	References
EMT	Pulmonary arterial hypertension	0.05 mg/kg/day	↓ PASMCs proliferation, ↓HIF‐1α and HIF‐2α, Rho‐kinase, ↓RV systolic pressure	[67]
EMT	Human cytomegalovirus	75 nM	↓RPS14, ↑MDM2, ↑ p53, ↓IE1/2, UL44 and pp65	[68]
EMT	COVID‐19	1 mg/kg	↓SARS‐CoV‐2 replication, ↓IL‐6, ↓TNF‐α	[69]
EMT	Neurophysiological effects	32 and 64 μg/μL, respectively	↓Amino acid incorporation, ↓Theta power	[70]
Emetine dihydrochloride	Dengue fever	0.01–10 μM	↓DENV replication, ↓RNA synthesis	[71]
EMT	Zika virus	2 mg/kg/day	↓NS1 protein, ↓ZIKV replication, ↓NS5 polymerase	[72]
	Ebola virus	1 mg/kg/day	↓Lysosomal function, ↓Viral entry	
EMT	Emetogenic action	0.03–1.0 mM	↑ Fos‐ir cells	[73]
Emetine dihydrochloride	Type 1 diabetes mellitus	0.002–2 mg/kg	↓TNF release	[74]
EMT	Enterovirus‐A71	0.20 mg/kg	↓Viral loads, ↓IRES translation	[75]

*Note:* ↑: increase/activation/upregulation/stimulation; ↓: decrease/inhibition/downregulation.

Abbreviations: HIF, hypoxia‐inducible factor; IL‐6, interleukin‐6; IRES, internal ribosome entry site–mediated translation; MDM2, mouse double minute 2 homologue; NS5, non‐structural protein 5; PAH, pulmonary arterial hypertension; PASMCs, pulmonary arterial smooth muscle cells; pp65, phosphoprotein 65; RPS14, ribosomal protein S14; RV, right ventricular; TNF, tumour necrosis factor; UL44, unique long 44.

### 3.3 Toxicological Profile of Emetine

Toxicological studies is an important paramount in the screening of newly developed drugs before it can be used on humans and carried out on experimental animals [76]. Natural products have biological or pharmacological activity that is useful in drug development and design but they can cause toxicity, which is a challenge in drug development [77]. The toxicity of EMT, like most drugs, is dose‐related [78]. An investigation found that, higher dose of EMT and dehydroemetine inducing cardiotoxicity due to I_CaL_ inhibition [79]. In another study found that treated with EMT dihydrochloride at 5.54 mg/kg induced nausea in rats [80]. Several studies have demonstrated that EMT and its derivatives shows cytotoxicity in various cell lines, including AML, PaCa3, PC3, DU145, LNCaP, A549 and H1299 [36, 81, 82, 83]. Although EMT exhibits cytotoxic effects against several cancer cell lines, its overall safety profile remains inadequately characterized and may limit translational applicability. Known adverse effects including cardiotoxicity, myopathy, and emetic responses at higher doses underscore the importance of rigorous dose optimization and toxicological profiling before advancing to clinical studies. Importantly, many available studies primarily focus on anticancer efficacy, while systematic long‐term toxicological evaluations remain scarce. This imbalance limits accurate assessment of the therapeutic index of EMT.

### 3.4 Clinical Evidence

Clinical trials involved the investigation of a new drug that are studies or trials on humans and also have the potential to pose unknown risks to their participants [84, 85]. However, natural products have long been a major source of potential drugs [86]. A trial involving 63 patients in China found that lower‐dose EMT administration was associated with improvement of clinical symptoms in a limited COVID‐19 cohort; however, the study size and safety assessment were insufficient to establish definitive clinical safety [87]. In a Phase I clinical trial, 32 patients who received EMT hydrochloride daily for 10 days reported toxicity like pain at the injection site, muscle weakness and myalgia, elevations in creatine phosphokinase and cardiac arrest [88]. A clinical trial in Thailand involving 100 patients found that combining mebendazole with EMT hydrochloride achieved a 70% cure rate for paragonimiasis [89]. Although several in vitro and in vivo studies have investigated the anticancer potential of EMT is performed but their clinical studies are limited. More preclinical and clinical evaluations are needed to determine its therapeutic potential for human therapy.

### 3.5 Novelty, Key Findings, Limitation and Future Perspective of This Study

The novelty of this study lies in demonstrating the ability of EMT derivatives to modulate the PI3K/AKT/mTOR pathway and a thorough assessment of its anticancer potential across various types of cancer. This review additionally delivers a detailed evaluation of dosing efficacy in both cell‐based and animal models which will serve as a foundation for subsequent clinical investigations. It also emphasizes different cancers like lung, brain, gastric. In contrast to earlier research that broadly investigated pharmacological effects in a general context, this review systematically compiles and analyses the toxicological profile, preclinical efficacy and clinical trial outcomes of EMT derivatives, focusing on their regulation of the PI3K/AKT/mTOR signalling pathway across multiple cancer types. Although EMT has demonstrated preclinical anticancer activity in both in vivo and in vitro, rigorous clinical trials are still lacking to substantiate its safety and efficacy in humans. Future studies should carefully evaluate whether EMT derivatives can achieve acceptable efficacy‐to‐toxicity ratios for potential therapeutic development. Furthermore, comprehensive toxicological evaluations and expansive clinical trials are necessary to validate their safety and therapeutic potential in targeting cancer.

## 4 Conclusion

In conclusion, the overall results highlight that EMT derivatives demonstrate encouraging preclinical anticancer activity, at least in part through inhibition of PI3K/AKT/mTOR signalling pathways, which are crucial in survival, tumour growth, angiogenesis and metastasis. These compounds exhibit broad biological activities, including antiviral, anticancer, antiparasitic and contraceptive activities. Although preclinical studies suggest preferential cytotoxicity toward cancer cells, the safety profile of EMT is not fully established. Dose‐dependent cardiotoxicity, emetic effects, and limited clinical data represent significant hurdles that must be addressed before clinical application can be considered. Despite promising experimental findings, the current evidence base remains insufficient for clinical translation. Future investigations should focus on pharmacokinetic optimization, molecular target validation, safety profiling, and well‐designed clinical trials before EMT‐based therapeutics can be considered for routine oncological application.

## Author Contributions

**Samy Selim:** validation, writing – review and editing, writing – original draft, visualization, software, resources, data curation, formal analysis. **Md. Sakib Al Hasan:** conceptualization, writing – original draft, writing – review and editing, formal analysis, project administration, supervision. **Asif Hassan Malik:** conceptualization, writing – original draft, writing – review and editing, visualization, validation, software, resources. **Mohammad Y. Alshahrani:** conceptualization, writing – original draft, writing – review and editing, visualization, validation, formal analysis, project administration, supervision. **Imam Hossen Rakib:** investigation, writing – original draft, writing – review and editing, formal analysis. **Md. Arif Hossain:** conceptualization, investigation, validation, methodology. **Emon Mia:** conceptualization, writing – original draft, writing – review and editing, methodology, software, formal analysis, data curation, resources. **Mst. Ismatara Khatun:** conceptualization, writing – original draft, writing – review and editing, formal analysis, software, validation, methodology, investigation, data curation, resources. **Abul Bashar Ripon Khalipha:** conceptualization, writing – original draft, writing – review and editing, formal analysis, supervision, project administration. **Anike Chakrabarty:** conceptualization, writing – original draft, writing – review and editing. **Noshin Tasnim Yana:** conceptualization, writing – original draft, writing – review and editing, visualization, software, resources.

## Funding

The authors have nothing to report.

## Conflicts of Interest

The authors declare no conflicts of interest.

## Data Availability

No primary data were generated or analysed in this study. The review is based exclusively on previously published literature, and all relevant sources are cited within the article.
